# In Vitro Evaluation of Neutral Aryloximes as Reactivators for *Electrophorus eel* Acetylcholinesterase Inhibited by Paraoxon

**DOI:** 10.3390/biom9100583

**Published:** 2019-10-08

**Authors:** Daniel A. S. Kitagawa, Samir F. de A. Cavalcante, Reuel L. de Paula, Rafael B. Rodrigues, Leandro B. Bernardo, Munique C. J. da Silva, Thiago N. da Silva, Wellington V. dos Santos, José M. Granjeiro, Joyce S. F. D. de Almeida, Marcos C. Barcellos, Ana Beatriz de A. Correa, Tanos C. C. França, Kamil Kuča, Alessandro B. C. Simas

**Affiliations:** 1 Laboratory of Molecular Modelling Applied to Chemical and Biological Defense (LMACBD), Military Institute of Engineering (IME), Praça General Tibúrcio 80, Rio de Janeiro 22290-270, Brazil; 2 Brazilian Army Institute of Chemical, Biological, Radiological and Nuclear Defense (IDQBRN), Brazilian Army Technological Center (CTEx), Avenida das Américas 28705, Rio de Janeiro 23020-470, Brazil; 3 Walter Mors Institute of Research on Natural Products (IPPN), Federal University of Rio de Janeiro (UFRJ), CCS, Bloco H, Rio de Janeiro 21941-902, Brazil; 4 Castelo Branco University (UCB), School of Pharmacy, Avenida Santa Cruz 1631, Rio de Janeiro 21710-255, Brazil; 5 Department of Chemistry, Faculty of Science, University of Hradec Králové, Rokitanskeho 62, 50003 Hradec Králové, Czech Republic; 6 National Institute of Metrology, Standardization and Industrial Quality (INMETRO), Avenida Nossa Senhora das Graças 50, Duque de Caxias 25250-020, Brazil; 7 Emergency and Rescue Department (DSE), Rio de Janeiro State Fire Department (CBMERJ), Praça São Salvador 4, Rio de Janeiro 22231-170, Brazil; 8 School of Biomedicine, University Universus Veritas (UNIVERITAS), Rua Marquês de Abrantes 55, Rio de Janeiro 22230-060, Brazil

**Keywords:** acetylcholinesterase, pesticides, neutral oximes, antidotes, drug design

## Abstract

Casualties caused by organophosphorus pesticides are a burden for health systems in developing and poor countries. Such compounds are potent acetylcholinesterase irreversible inhibitors, and share the toxic profile with nerve agents. Pyridinium oximes are the only clinically available antidotes against poisoning by these substances, but their poor penetration into the blood-brain barrier hampers the efficient enzyme reactivation at the central nervous system. In searching for structural factors that may be explored in future SAR studies, we evaluated neutral aryloximes as reactivators for paraoxon-inhibited Electrophorus eel acetylcholinesterase. Our findings may result into lead compounds, useful for development of more active compounds for emergencies and supportive care.

## 1. Introduction

**Scheme 1:**
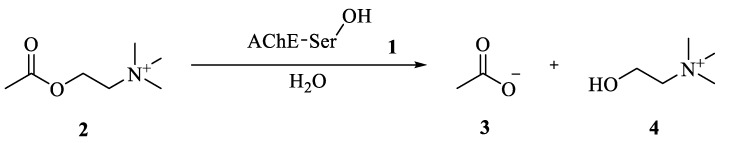
Hydrolysis of ACh by AChE.

Acetylcholinesterase (AChE, EC 3.1.1.7, **1**) is a serine-esterase, a key enzyme for the parasympathetic neurotransmission. This enzyme can be found in the brain, erythrocytes, and muscles, being responsible for the hydrolysis of the neurotransmitter acetylcholine (ACh, **2**) into its two precursors, acetate (**3**) and choline (**4**), ending the potential action at the post-synaptic cleft (Scheme 1) [1,2,3,4]

**Figure 1:**
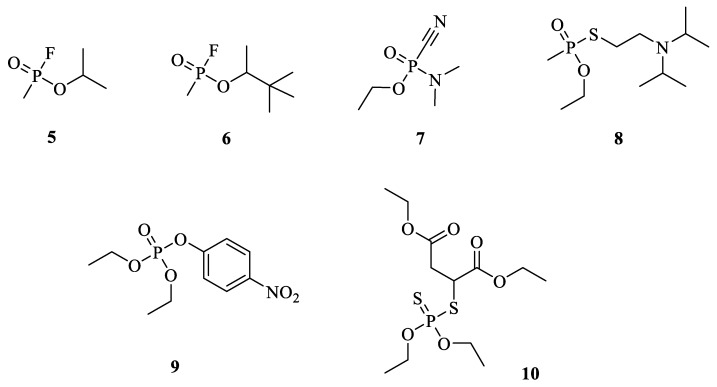
Toxic organophosphorus compounds.

The hydrolysis of ACh by AChE is accomplished at the esteratic site (a catalytic triad composed by residues serine, histidine, and glutamate), which is highly conserved throughout the species. Inhibition of the catalytic serine residue via phosphorylation leads to accumulation of ACh, causing overstimulation of cholinergic innervations and ultimately, death [5,6,7,8]. Organophosphorus (OP) compounds are well-studied AChE inhibitors. The nerve agents sarin (**5**), soman (**6**), tabun (**7**), VX (**8**), and pesticides such as paraoxon (**9**) and malathion (**10**), are relevant examples of such compounds (Figure 1) [8,9]. While nerve agents are strictly regulated by the Chemical Weapons Convention (CWC) [10], a multilateral treaty that entered into force in 1997, pesticides are not regulated by any international agency, and their use is still subjected to each country’s discretion. Consequently, non-developed and even developing countries still use pesticides already forbidden in the United States and Europe [11,12,13,14]. Recently, the Brazilian Parliament has discussed a controversial lift of the ban for using some aggressive pesticides, raising fears of pollution of lands, water courses, and groundwater, as well as the intoxication of workers and animals [15,16,17,18]. Besides economic impact in trade agreements, environmental and occupational issues, accidental poisoning of children, and suicide attempts are also additional costs for the governmental health systems [8,19,20,21,22,23,24,25,26,27]. These harmful chemicals are also associated with neurodegenerative diseases [28].

**Scheme 2:**
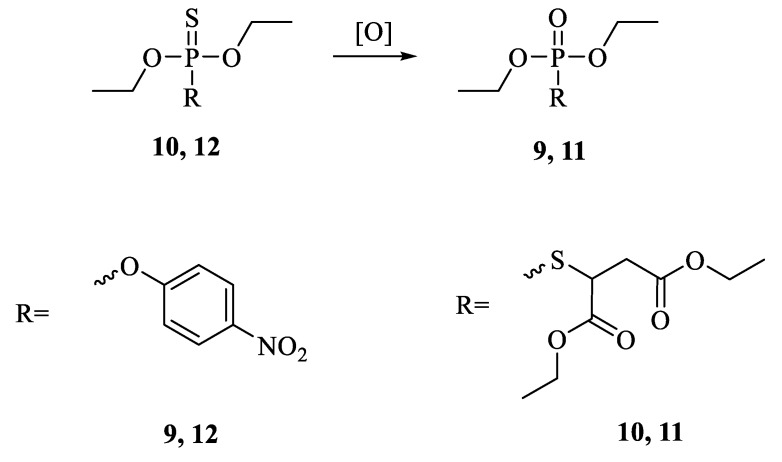
Conversion of thions to oxons by oxidases.

Aiming to control mosquito vectors in densely populated areas of the tropical countries, insecticides have been aerosolized. Despite of the inefficiency of this methodology and studies accounting for development of vector’s resistance, it is still used. Tropical diseases are a burden for health systems in developing and under-developed countries. For example, arboviruses in Brazil transmitted by *Aedes aegypti* (Dengue, Yellow Fever, Zika, and Chikungunya) and *Anopheles* sp. (Malaria) have high rate of mortality and morbidity, among other mosquito-borne diseases. Brazilian National Health System (*Sistema Único de Saúde*—SUS) and other ministries have spent large sums to try to control these vectors and treat affected people. In Brazilian cities, an aerosol dispositive (in Brazilian Portuguese, this aerosol is known popularly as “*fumacê*”) which contains malathion (**10**) has been used. However, it is a non-selective pesticide, leading to not only resistance, but also accidental and occupational poisonings, which may cause additional costs to SUS. Besides, it is also related to elimination of pollinator species. Malathion is in vivo converted to a more toxic metabolite, malaoxon (**11**). Parathion (**12**) reacts similarly with oxidases to yield the more toxic compound, paraoxon (**9**, Scheme 2) [29,30,31,32,33,34].

**Scheme 3:**
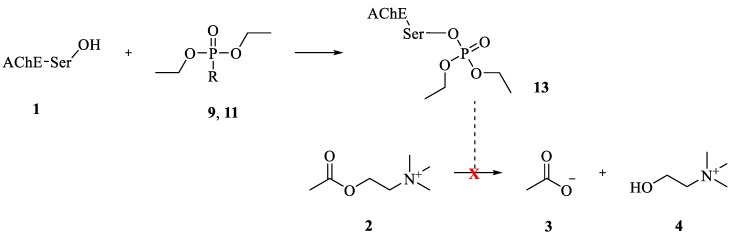
AChE inhibition by oxons.

Pesticides oxo-forms [Oxons (**9**, **11**)] are more toxic than their thio forms [thions (**10**, **12**)], although both are able to phosphorylate the catalytic serine residue in AChE (**1**), bringing about ACh (**2**) accumulation. Thio forms are converted slowly in the organism to more toxic oxo forms. This process is called “lethal synthesis” [35]. Depending on the level of exposure, poisoning may be fatal due to the *SLUDGEM* syndrome (salivation, lacrimation, urination, defecation, gastrointestinal disturbs, emesis, miosis, and muscle spasms). Scheme 3 shows a representation of reaction between an oxon (**9**, **11**) and AChE (**1**), yielding an oxon-AChE adduct [36].

Intoxication caused by oxons must be rapidly addressed. Treatment usually employs a combination of up to three different drugs: an enzymatic reactivator, to displace the organophosphorus moiety from the serine residue (currently pyridinium oximes), an anticholinergic agent, to reduce the cholinergic stimulus, and an anticonvulsant, to control seizures. Delays in medical response may lead to AChE reactivators being ineffective, as excitatory neuronal mechanisms take over. This event has brought about the use of GABA agonists and glutamate antagonists in organophosphorus poisoning. Depending on the agent, enzymatic aging may occur, rendering no possibility of AChE reactivation with therapeutically available compounds, due to the formation of a stable phosphate ion between phosphorylated serine and protonated histidine at esteratic site [37,38,39,40,41].

**Figure 2:**
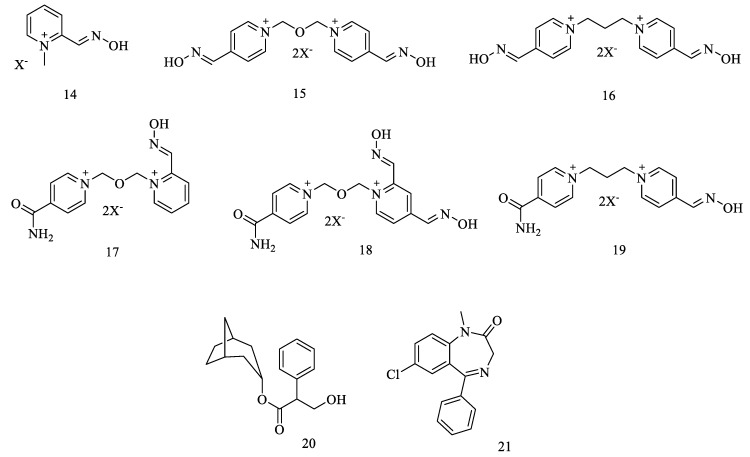
Structures of compounds used for organophosphate poisoning.

As we mentioned above, to this date, pyridinium oximes are the only clinically available AChE reactivators for organophosphate poisoning. Pralidoxime (2-PAM, **14**, X^−^ is either chloride, iodide, or mesylate), obidoxime (**15**, X^−^ is chloride), trimedoxime (TMB, **16,** X^−^ is bromide), HI-6 (**17,** X^−^ is chloride), HLö-7 (**18,** X^−^ is chloride), and K027 (**19,** X^−^ is bromide) are examples of active compounds [18,41,42]. At physiological pH, they are converted to oximates, nucleophilic species that displace the OP moiety, reactivating the enzyme. Atropine (**20**) and diazepam (**21**) are the other components of antidote kit, as anticholinergic and anticonvulsant drugs, respectively (Figure 2).

**Scheme 4:**
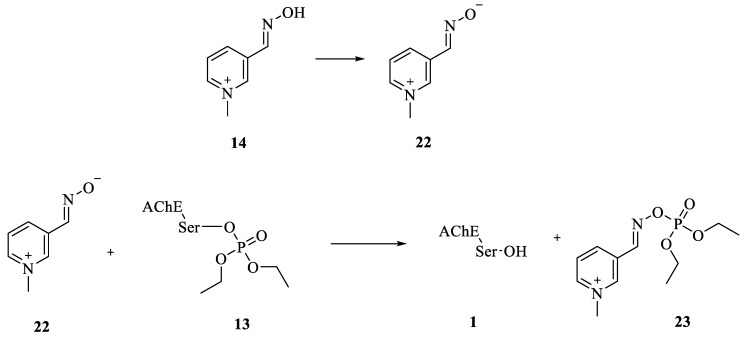
Reactivation of AChE by pyridinium oximates.

Scheme 4 represents the reaction of pralidoxime oximate (**22**) with an oxon-AChE adduct (**13**). Although they are the only current class available for clinical use, their permeability through the blood-brain barrier is limited, due to their positive charge, reducing their effectiveness at the central nervous system (CNS). Therefore, the development of molecules with improved physicochemical profile, able to reach higher concentrations in the brain for better and fast AChE reactivation, is warranted. Moreover, it is noteworthy that there is no universal antidote for the AChE irreversible inhibitors [43,44,45,46].

A number of research endeavors have aimed at the improvement of existing antidotes [47,48,49,50,51,52,53,54]. One of the limitations of current AChE reactivators is their ability to cross the blood-brain barrier (BBB), which reduces the amount of reactivator in the brain. The main goal of the present work was the evaluation of simple, neutral aryloximes as reactivators for paraoxon-inhibited AChE using Ellman’s spectrophotometric assay and commercial *Electrophorus eel* as a source of AChE, a model of study due to full homology of its active site in comparison to the human isoform, although some relevant differences have been noted [55,56,57]. Thus, we chose to assay simple non-cationic aryloximes, substituted with electron withdrawing or donating groups for new insights on structural requirements to prospective novel lead compounds with enhanced pharmacokinetics [46,49,58].

## 2. Experimental

### 2.1. General Information

Syntheses of all neutral aryloximes and reference compounds obidoxime dichloride and trimedoxime dibromide have been detailed in our previous paper, with yields varying from 40 to 98% of pure products [55]. Their purity was checked using TLC-MS, GC-MS, and LC-MS before assays. Acetylthiocholine iodide (ATCI), paraoxon-ethyl 90%, 5,5′-dithiobis-(2-nitrobenzoic) acid (DTNB), lyophilized acetylcholinesterase from *Electrophorus eel* (*Ee*AChE, 1000U per mg protein, type V-S, C2888), pralidoxime iodide (2-PAM), dimethyl sulfoxide (DMSO, biological grade, dry, oxygen-free sealed bottle), sodium hydroxide (pellets), sodium phosphate monobasic hydrate, and sodium phosphate dibasic dihydrate were purchased from Sigma-Aldrich (São Paulo, Brazil). Absolute ethanol was purchased from Tedia (Rio de Janeiro, Brazil). Purified water was obtained from Millipore Milli-Q system (18.2 MΩ cm at 25 °C, Millipore Brazil, São Paulo, Brazil). TLC (Thin Layer Chromatography) aluminum plates coated with silica gel F_254_ were purchased from Merck Brazil (São Paulo-SP, Brazil). Camag TLC-MS (Thin Layer Chromatography-Mass Spectrometry) interface was used to follow reactions (AuTeC, São Paulo, Brazil). GC-MS (Gas Chromatography-Mass Spectrometry) data were obtained from Agilent 6890 GC system equipped with 5975C mass spectrometer detector (Billerica, Massachusetts, USA). LC-MS (liquid chromatography—mass spectrometry) data were obtained from Agilent 1210 LC system equipped with 6410B triple quadrupole mass spectrometer detector (Billerica, Massachusetts, USA). SpectraMax Plus 384 microplate reader (Molecular Devices, San Jose, California, USA) was used in all assays. Kasvi 96-wells microplates were purchased from Kasvi Brasil (São José dos Pinhais, Paraná, Brazil). Gilson single channel pipettes were purchased from Gilson Inc. (Middleton, Wisconsin, USA) and Eppendorf 8-channel pipettes were acquired from Eppendorf Brasil (São Paulo-SP, Brazil). Ellman’s tests [59] were performed in triplicate, at three different assays, by at least three different operators, measured at 24 °C ± 2 °C. Microsoft Excel 2010^®^ was used for all calculations. All disposable materials and glassware in contact with paraoxon were decontaminated with aqueous solution containing 10% w/v NaOH and 10% w/v NaClO (pH = 14) for 48 h at room temperature before correct disposal. Estimations of pKa and logP for reference antidotes and test compounds were obtained from ChemAxon Online Suite (chemicalize.org).

### 2.2. Preparation of Test Solutions

Fresh solutions of paraoxon (final concentrations in wells from 10^−3^ to 10^−9^ mol/L) were prepared by dissolving the commercial standard in absolute ethanol and stored at −20 °C until use. Stock solutions of test oximes and clinical references (10^−2^ mol/L) were prepared by dissolution in DMSO, and phosphate buffer solution (PBS, pH 7.60 ± 0.10) was added to prepare test solutions (final concentrations in wells 1000, 100 and 10 µmol/L). They were sonicated for 5 min before use. During experiments, all solutions were kept at 0 °C. DMSO did not affect measurements in our conditions [60].

### 2.3. Ellman’s Spectrophotometric Assays

Ellman’s assay was used for determination of optimal paraoxon concentration and reactivation level. It was performed in accordance to our previously published procedure [60], using 96-wells microplates (final volume 200 µL). For AChE inhibition, we adjusted the microplate reader to 412 nm, a wavelength at which the acetylthiocholine-DTNB adduct absorbs, and pipetted 70 µL of *Ee*AChE 2.14 U/mL (prepared from commercial lyophilized), 80 µL of DTNB 0.4 mg/mL, 20 µL of PBS, 10 µL of paraoxon solution (positive control, A_i_; CAUTION as paraoxon is a potent cholinesterase inhibitor) or 10 µL of PBS (negative control, A_0_), incubating for 10 min for inhibition reaction. Then, we added 20 µL of ATCI 1 mmol/L and read the absorbance in different times (0, 15, 30, and 60 min) to calculate enzyme inhibition (A_i_). AChE inhibition percent was calculated using Equation (1).

(1) $$\%I=100\times \frac{A_{0}-A_{i}}{A_{0}}$$

For AChE reactivation using oximes, we adjusted microplate reader to 412 nm and pipetted 70 µL of *Ee*AChE 2.14 U/mL, 80 µL of DTNB 0.4 mg/mL, 10 µL of inhibitor. After inhibition reaction (10 min), we added 20 µL standard antidotes or test molecules in different concentrations and waited for 30 min for reactivation reaction. At last, we pipetted 20 µL of ATCI 1 mmol/L and read the absorbance (A_r_) in different times (0, 15, 30, and 60 min) to calculate enzyme reactivation. AChE reactivation percent was calculated using Equation (2).

(2) $$\%R=100\times \frac{A_{r}-A_{i}}{A_{0}-A_{i}}$$

## 3. Results and Discussion

To determine the highest concentration of paraoxon to be used in our Ellman’s conditions without causing full inhibition of *Ee*AChE, which may lead to inconsistent results, we set forth the inhibition assay with paraoxon final concentrations ranging from 10^−3^ to 10^−9^ mol/L (in ethanol), setting 10 min as the inhibition time. We also intended to determine the detection limit for our method using paraoxon as *Ee*AChE inhibitor.

After 10 min of incubation of the enzyme with organophosphate, we read the absorbance immediately after the addition of ATCI and at each 15 min (15 to 60 min). The highest inhibition achieved was 92.5 and 93.3%, at 10^−4^ and 10^−5^ mol/L, respectively, after 15 min of addition of substrate. These values were virtually the same during all measured times (30 and 60 min). Based on the results, for all reactivation experiments we opted for 10^−5^ mol/L as paraoxon concentration, to ensure safety to all operators during the experiments (inhibition data available in Table S1). Paraoxon concentrations of 10^−7^ to 10^−9^ mol/L led to inconsistent results, confirming that 10^−5^ mol/L is the limit of detection for our assay (final concentrations of *Ee*AChE and ATCI, 10^−4^ mol/L and 2.14 U/mL, respectively), as previously reported [61].

**Scheme 5:**
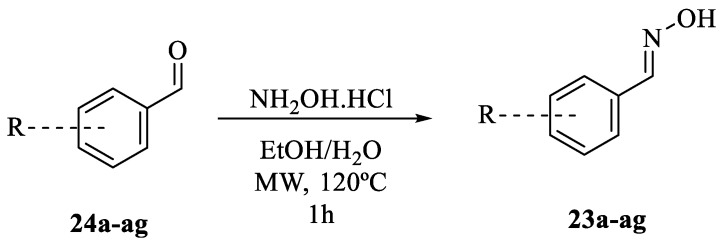
Synthesis of aryloximes.

Table 1 lists all 33 neutral aryloximes tested (**23a**–**ag**), their estimated properties, and reactivation percent at different concentrations. Synthesis of tested compounds from related aldehydes (**24a**–**ag**) using microwave irradiation has been described in our previous paper [55], in accordance with Scheme 5.

Table 1 shows that all compounds have higher calculated logP values when compared to than the standard antidotes used (entries 34–36). Higher logP values for the neutral oximes are expected to improve the ability of these simple molecules to cross the blood-brain barrier when compared with clinically available compounds. Presence of reactivator in CNS is necessary to exert central action [62,63]. Analysis of calculated pKa of tested compounds and comparison to clinical compounds showed similar pattern. SAR studies indicate that ideal pKa values are between 7.0 and 9.0, suggesting that some compounds may be relevant for further synthetic improvement. Neutral aryloximes synthesized were evaluated as reactivators for paraoxon-inhibited *Ee*AChE in three different concentrations. Although some neutral oximes had been previously tested using blood samples (entries 6, 19, 26, and 31) [58], we decided to include them for comparison using our procedure.

2-PAM (**14**, entry 34) was selected for direct comparison due to structural similarity; obidoxime (**15**, entry 35) and trimedoxime (**16**, entry 36), bispyridinium compounds are more effective in AChE reactivation, and were also evaluated for further analysis. All compounds were screened at 412 nm to verify possible absorbance and oximolysis, i.e., reaction with Ellman’s reagents. These values were deducted to retrieve confident values. Absorbance values were obtained after 30 min of incubation of test compounds with paraoxon-inhibited *Ee*AChE. We also set as threshold 10 ± 1% of reactivation of paraoxon-inhibited *Ee*AChE to select compounds for further structural modification. We defined this value as the minimum score in accordance with the literature [64].

Literature reports that the maximum concentration tolerated in vivo for clinical compounds, pyridinium oximes, is 100 µmol/L [64,65]. Neutral aryloximes lack of key structural motifs, for instance, cationic nitrogen for interaction with the catalytic anionic site of AChE. To evaluate the effect of concentration on EeAChE reactivation, we tested all neutral aryloximes at 1000, 100, and 10 µmol/L. Reactivation at the highest concentration was achieved for some compounds, but they had no reactivation at 10 µmol/L, whatsoever.

3-Oximes from isatin (entry 32) and N-benzylisatin (entry 33) were the only tested compounds to achieve the threshold at 100 µmol/L, with isatin-3-oxime reactivating AChE more effectively than N-benzyl derivative. We speculate this outcome might be due to the steric hindrance of benzyl group, making more difficult the approach of the oximate to the phosphorus atom. Nonetheless, at 1000 µmol/L, N-benzyl isatin 3-oxime was slightly more active than non-benzylated analogue. Further studies, including in silico approaches, should be done in order to rationalize these results. Isatin derivatives have also previously been described as cholinesterase inhibitors, indicating that this motif may be a starting point for synthesis of more active compounds [66,67,68,69,70,71,72].

At 1000 µmol/L, we could identify nine additional compounds. Salicylaldoxime (2-hydroxybenzaldoxime, entry 1) presented some reactivation ability, as we hypothesize a positive effect by the neighboring hydroxyl group, contributing either to oximate generation or to its nucleophilicity [47]. Satisfyingly, this is an accessible compound which may be synthetically manipulated for optimization. 2-Chlorobenzaldoxime (entry 10) also presented good activity. Recent literature showed that this structural motif has been able to reactivate AChE inhibited by nerve agents [73,74]. All three trifluoromethyl-substituted benzaldoximes (entries 16–18) showed relevant reactivation of paraoxon-inhibited EeAChE. Interestingly, the 3-trifluoromethyl compound performed much better than 2- and 4- isomers, not in parallel with the calculated pKa, lower in 2-substituted isomer (Table 1, entries 16–18). We postulated that for 3-substituted compound, the polar hydrophobicity exerted by the C-F bonds, largely explored in medicinal chemistry, might play a role [75,76]. 3-methyl and 4-methylbenzaldoximes (entries 20 and 21, respectively) also showed good performance in reactivation of paraoxon-inhibited EeAChE. Conversely, the lower activities of 2-methyl isomer (entry 19) and 4-isopropylbenzaldoxime (entry 22) might be explained, respectively, by steric effects that affect the oximate attack proper interaction inside the active site. Isovanillin (entry 28) and, markedly, orthovanillin (entry 29) also performed satisfactorily. Neutral pyridine-4-aldoxime (entry 30) and pyridine-2-aldoxime (entry 31) exhibited significantly lower reactivation potency compared to 2-PAM, although they have structural similarity. This indicates the importance of quaternary nitrogen atom for development of active compounds [44].

## 4. Conclusions

In conclusion, after we surveyed across a series of simple, neutral oximes, in order to identify structures for further synthetic improvement, we could identify 12 substances whose motifs can be incorporated in development of lead compounds by analysis of different substitution patterns in order to address poisoning with paraoxon. We observed that either electron-donating or electron-withdrawing groups could not only bestow enhanced pharmacokinetics (pKa, logP), but also reactivation potency. We are now testing the same compounds with other AChE isoforms for screening of compounds that may be useful for further development of novel antidotes for other pesticides and nerve agents’ surrogates, and also AChE inhibitors.

## Figures and Tables

**Table 1:** Oximes **22a**–**ag** synthesized for assay in this work.

Entry	Code	Name	pKa ^1^	logP	Reactivator Concentration (µmol/L)		
					1000	100	10
1	22a	2-hydroxybenzaldoxime	6.61 (OH = 9.99)	1.39	9 ± 1	4 ± 1	1 ± 0
2	22b	3-hydroxybenzaldoxime	7.09 (OH = 9.74)	1.39	3 ± 0	3 ± 0	2 ± 0
3	22c	4-hydroxybenzaldoxime	7.57 (OH = 10.15)	1.39	6 ± 1	6 ± 1	6 ± 1
4	22d	2-methoxybenzaldoxime	6.69	1.54	2 ± 0	2 ± 0	1 ± 0
5	22e	3-methoxybenzaldoxime	7.20	1.54	4 ± 0	1 ± 0	1 ± 0
6	22f	4-methoxybenzaldoxime	7.69	1.54	4 ± 1	2 ± 0	2 ± 0
7	22g	2-bromobenzaldoxime	6.96	2.46	3 ± 0	2 ± 0	2 ± 0
8	22h	3-bromobenzaldoxime	7.31	2.46	4 ± 0	1 ± 0	0
9	22i	4-bromobenzaldoxime	6.69	2.46	2 ± 0	2 ± 0	2 ± 0
10	22j	2-chlorobenzaldoxime	6.80	2.30	30 ± 2	7 ± 1	2 ± 0
11	22k	3-chlorobenzaldoxime	7.26	2.30	3 ± 0	2 ± 0	1 ± 0
12	22l	4-chlorobenzaldoxime	6.67	2.30	3 ± 0	3 ± 0	1 ± 0
13	22m	2-fluorobenzaldoxime	6.82	1.84	4 ± 0	1 ± 0	3 ± 0
14	22n	3-fluorobenzaldoxime	7.11	1.84	4 ± 0	2 ± 0	2 ± 1
15	22o	4-fluorobenzaldoxime	6.80	1.84	3 ± 0	2 ± 0	2 ± 0
16	22p	2-trifluoromethyl benzaldoxime	5.52	2.57	26 ± 3	5 ± 0	1 ± 0
17	22q	3-trifluoromethyl benzaldoxime	6.13	2.57	77 ± 4	5 ± 0	1 ± 0
18	22r	4-trifluoromethyl benzaldoxime	6.29	2.57	10 ± 1	4 ± 0	1 ± 0
19	22s	2-methylbenzaldoxime	8.08	2.21	6 ± 0	1 ± 0	1 ± 0
20	22t	3-methylbenzaldoxime	7.97	2.21	12 ± 2	1 ± 0	0
21	22u	4-methylbenzaldoxime	8.14	2.21	14 ± 2	0	0
22	22v	4-isopropylbenzaldoxime	8.21	2.94	2 ± 0	1 ± 0	2 ± 0
23	22w	3-nitrobenzaldoxime	5.83	1.64	6 ± 0	3 ± 0	5 ± 1
24	22x	4-nitrobenzaldoxime	5.80	1.64	8 ± 1	7 ± 1	4 ± 1
25	22y	4-(N,N-dimethylamino) benzaldoxime	8.71	1.80	7 ± 1	6 ± 0	2 ± 0
26	22z	4-(N,N-diethylamino) benzaldoxime	8.80	2.52	7 ± 1	5 ± 1	5 ± 1
27	22aa	Vanillin oxime	7.18 (OH = 10.60)	1.23	5 ± 0	4 ± 0	4 ± 1
28	22ab	Isovanillin oxime	6.21 (OH = 10.47)	1.23	10 ± 0	4 ± 0	4 ± 0
29	22ac	Orthovanillin oxime	7.18 (OH = 10.14)	1.23	22 ± 2	4 ± 0	4 ± 0
30	22ad	Pyridine-4-aldoxime	10.21	0.48	6 ± 1	5 ± 0	1 ± 0
31	22ae	Pyridine-2-aldoxime	9.02	1.15	9 ± 1	3 ± 0	0
32	22af	Isatin 3-oxime	7.13 (NH = 15.51)	0.96	67 ±10	15 ± 2	8 ± 0
33	22ag	N-benzylisatin 3-oxime	7.31	2.55	85 ±10	9 ± 1	1 ± 0
34	14	Pralidoxime (2-PAM) ^2^	7.63	-3.26	39 ± 2	42 ± 2	16 ± 3
35	15	Obidoxime (OBD)	7.51, 8.11	-6.93	62 ± 3	88 ± 3	58 ± 3
36	16	Trimedoxime (TMB)	8.63, 9.24	-7.04	84 ± 5	75 ± 3	29 ± 2

**^1^** pK values refer to the dissociation Ar-C=N-OH ⇌ Ar-C=N-O^−^ + H^+^ unless otherwise indicated. **^2^** Commercial source.

## Supplementary Materials

The following are available online at https://www.mdpi.com/2218-273X/9/10/583/s1. Table S1: Full results on inhibition of the *Ee*AChE with paraoxon
