# Supplementary material for: In Vitro Evaluation of Neutral Aryloximes as Reactivators for Electrophorus eel Acetylcholinesterase Inhibited by Paraoxon
Source: Biomolecules. 2019 Oct 8;9(10):583. doi: 10.3390/biom9100583 (PMC6843506; doi:10.3390/biom9100583)
Supplement: Supplementary file 1 [file biomolecules-09-00583-s001.pdf]

Supplementary Material

Table S1. Percent of *Ee*AChE inhibition with POX.

**POX Concentration (mol/L)**	**% *Ee*AChE Inhibition**
10^{-9}	5.1
10^{-8}	5.0
10^{-7}	1.7
10^{-6}	22.4
10^{-5}	93.3
10^{-4}	92.5
10^{-3}	72.1
